# Antiplasmodial Activity and Cytotoxicity of Plants Used in Traditional Medicine of Iran for the Treatment of Fever

**Published:** 2015

**Authors:** Somayeh Esmaeili, Azadeh Ghiaee, Farzaneh Naghibi, Mahmoud Mosaddegh

**Affiliations:** a *Department of Pharmacognosy,School of Pharmacy, Shahid Beheshti University of Medical Sciences, Tehran, Iran. *; b *Traditional Medicine and Materia Medica Research Centre (TMRC), Shahid Beheshti University of Medical Sciences. Tehran, Iran. *; c *Department of Traditional Pharmacy, School of Traditional Medicine, Shahid Beheshti University of Medical Science, Tehran, Iran.*

**Keywords:** * Rosa damascene*, Antiplasmodial, Malaria, Fever, Traditional medicine

## Abstract

Malaria is the most serious parasitic disease and one of the oldest recorded diseases in the world. Because of the resistance of malaria parasites to current drugs, it is necessary to discover new antiplasmodial drugs. Traditional medicine is one of the important sources of new antiplasmodial drugs.

In this study, twenty methanolic extracts from different parts of sixteen medicinal plants used in traditional medicine of Iran for the treatment of “*Nobeh fever*” and/ or fever were screened for *in-vivo *antiplasmodial activity against *Plasmodium berghei* and cytotoxic effect on Madin–Darby bovine kidney cells (MDBK).

Eleven species (55%) were found to have antiplasmodial activity. Methanolic extract from *Rosa damascena *Mill. reduced parasitemia by 57.7% compared to untreated control mice at intra-peritoneal (*i.p*.) injection doses of 10 mg/Kg per day for 4 days. This is the first report that mentioned *in-vivo *antiplasmodial activity of *Rosa damascena *Mill.

## Introduction

Malaria is the most serious parasitic disease and one of the oldest recorded diseases in the world. The name "mal" "aria"(meaning "bad air" in Italian) comes from 18^th^ century Italian word (1). It affects 219 million people per year worldwide and is the cause of 660,000 deaths per year (2).

In recent years, the increasing resistance of malaria parasites to drugs or malaria vectors to pesticides, led to discover and develop new antiplasmodial and new pesticides agents (1, 3). Traditional medicines have been used to treat malaria for thousands of years and are the source of the two main groups (artemisinin and quinine derivatives) of modern antiplasmodial drugs (3). The vast majority of people on this planet still rely on their traditional materia medica for their everyday health care needs (4). Besides, herbal medicines are widely believed to be safe and efficacious. Therefore, the potential of plants traditionally used to inhibit parasite growth without host toxicity must be assured (5). One of the methods which are applied for the safety evaluation is *in-vitro* cytotoxicity assay against normal cells (6).

Tertian, quartana and tropical are three different forms of malaria according to the type of parasite and period of fever (7). The term of malaria did not exist in the ancient medicinal books but ancient physicians knew this disease. Avicenna, the Iranian philosopher and physician, (980-1037AD) about 1000 yr ago described the clinical features of an intermittent febrile attack with 4-12 h period of cold, hot, and sweating stages which is actually the characters of paroxysm of malaria (8). Among the symptoms of malaria, fever attacks are the most common symptom. Physicians of traditional medicine were divided fever into three categories: “*Yomiyyeh fever* (ephemeral fever)” “*Degh fever* (hectic fever)” “*Ofouni fever *(infectious fever)”. Infectious fever occurs when *Akhlat* (structural components) receive external heat. *Ofouni fever* is periodical like malaria. Physician of traditional medicine said *“Nobeh fever”* to this fever (9).

In the present study, the *in-vivo *antiplasmodial and *in-*vitro cytotoxic effect of 20 extracts from 16 medicinal plants that were used in traditional medicine of Iran against “*Nobeh fever*” and other fevers (10-17) were evaluated.

## Experimental

*Plants selection*

Fever attacks are the most common symptoms of malaria. So, the term “fever” was searched in the ancient medicinal books of Iran for selecting the plants. 

*Preparation of plant samples*

Seven plant species out of sixteen plants used for treating *nobeh fever* and other fevers were collected from different places of Iran. The others were purchased from herbal market (Attari) (Table 1). The plants were identified by the taxonomist and voucher specimens were deposited at Traditional Medicine and Materia Medica Research Center (TMRC) herbarium.

*Plant extraction*

The selected parts of the collected plants were air-dried in shadow. Plants were crushed into powder using a hammer mill and extracted by maceration of 10 g of powdered dried material in methanol at room temperature for 24 h with constant shaking. The filtrates were concentrated to dryness by means of a rotary evaporator and used for antiplasmodial and cytotoxicity tests.

**Table 1:** Selected plants from the ancient medicinal books of Iran for antiplasmodial and cytotoxic assays.

**No.**	**Scientific name /Persian name**	**Family**	**Some traditional uses**	**Voucher**
1a	*Alhagi camelorum *Fisch./Taranjebin	Fabaceae	Cough, burning fevers(10-13,16)*	116-HMS
1b	*Alhagi camelorum *Fisch./Taranjebin	Fabaceae	Cough, burning fevers(10-13,16)	3258-TMRC
2	*Althaea officinalis *L./Khatmi	Malvaceae		115-HMS
3	*Bambusa arundinacea *Retz. /Tabaashir	Poaceae	Hemorrhoid, fevers(15)	118-HMS
4	*Cassia angustifolia *Vahl./Sanaa or Senaa	Fabaceae	Laxative, epilepsy, fevers(10)	113-HMS
5	*Carthamus tinctorius *L./Kajireh	Asteraceae	Phlegmatic fever, melancholia, dropsy(16-17)	1234-TMRC
6a	*Cichorium intybus *L./Kaasni	Asteraceae	Jaundice, quartan fever(12,15-16)	110-HMS
6b	*Cichorium intybus *L./Kaasni	Asteraceae		109-HMS
7	*Convolvulus scammonia *L./Saghmouneya	Convolvulaceae	Abortion, antihelminthic, fevers(10-12,16)	112-HMS
8	*Cotoneaster nummularia *Fisch. &Mey. /Shir khesht	Rosaceae	Fever, cough, jaundice(10,13-16)	1248-TMRC
9a	*Cordia myxa *L./Sepestaan	Boraginaceae	Expectorant, burning fevers(10,13,16-17)	111-HMS
9b	*Cordia myxa *L./Sepestaan	Boraginaceae		1379-TMRC
10	*Fumaria parviflora *Lam./Shaahtare	Fumariaceae	Jaundice, fever, blood purifier(10)	114-HMS
11	*Hedera helix *L. /Lablaab	Araliaceae	Quartan fever, colic, cough(10,17)	3720-TMRC
12	*Plantago psyllium *L./Esfarze	Plantaginaceae	Laxative, burning fevers, cough, gout(10,13-14)	117-HMS
13	*Portulaca oleracea *L./Khorfe	Portulacaceae	Migraine, burning fevers, hemorrhoid(13,15-16)	119-HMS
14	*Rosa damascena *Mill./Gole sorkh	Rosaceae	Headache, laxative, quartan fever(16)	1489-TMRC
15a	*Viola odorata *L./Banafshe	Violaceae	Burning fevers, cough, pleuritis(10,13-14,16)	121-HMS
15b	*Viola odorata *L./Banafshe	Violaceae		1467-TMRC
16	*Ziziphus jujuba *Mill./Onnab or Annab	Rhamnaceae	Cough, inflammations, tertian fever(10,12)	120-HMS

*: The number of references

*Biological assays*

*In-vivo antiplasmodial assay*

To carry out the screening of the extracts, the Peters’ 4-day suppressive test against *Plasmodium berghei *infection in mice was employed (18-21). All the procedure was accepted by Shahid Beheshti University Ethics Committee and in accordance with the principles for laboratory animal use and care in the European Community guidelines. On day 1 (D_0_), all experimental adult male albino mice weighing 20–25 g were infected by intra-peritoneal (*i.p*.) injection with 1×10^7^ infected erythrocytes. The mice were randomly divided into groups of five per cage and treated during consecutive days with 10 mg/Kg of the sample by *i.p* injection for 4 days (on days D_0_, D_1_, D_2_ and D_3_). Two control groups were used in this experiment, one treated with chloroquine as a positive control while the other group was kept untreated as a negative control. On day 5 (D_4_) of the test, thin blood smears were prepared and blood films were fixed with methanol. The blood films were stained with Giemsa, and then microscopically examined. Percentage of parasitaemia was counted based on infected erythrocytes calculated per 1000 erythrocytes.

*In-vitro cytotoxic assay*

Methanolic extracts of all plants were screened for cytotoxic with the Madin–Darby bovine kidney normal cells (MDBK). Suspension containing 1×10^4^ cell/mL was seeded into 96-well micro plates. After 24 h, cells were washed and maintained with different concentrations of extract for 3 days, at 37 ^◦^C, under 5% CO_2_ atmosphere. The initial concentration of extracts was 100 µg/mL in DMSO, which was serially diluted in complete culture medium with two fold dilutions to give six concentrations (100 – 3.125 µg/mL). The cytotoxicity of the plant extracts was determined using the colorimetric methylthiazole tetrazolium (MTT) assay (19-23) and scored as a percentage of absorbance reduction at 570 nm of treated cultures versus untreated control cultures. IC_50_ values on cell growth were obtained from the drug concentration–response curves. 5-Fluorouracil (5-Fu) was examined as a positive control.

**Table 2:** *In* *-* *vivo* antiplasmodial activity and *in-vitro *cytotoxic assays of the selected plants

No.	Scientific name	Plant part	%Suppression	Cytotoxicity, IC_50_ (µg/mL)
**1a**	*Alhagi camelorum* Fisch.	Manna	-55.5	>100
**1b**	*Alhagi camelorum* Fisch.	Whole plant	0.6	>100
**2**	*Althaea officinalis* L.	Flowers	4.2	>100
**3**	*Bambusa arundinacea* Retz.	Gum	26.0	NA
**4**	*Cassia angustifolia* Vahl.	Leaves	37.5	>100
**5**	*Carthamus tinctorius* L.	Aerial part	42.3	>100
**6a**	*Cichorium intybus* L.	Roots	-6.3	>100
**6b**	*Cichorium intybus* L.	Aerial part	-44.0	>100
**7**	*Convolvulus scammonia* L.	Gum resin	-29.8	38.86
**8**	*Cotoneaster nummularia *Fisch. & Mey.	Fruit-bearing branches	41.9	>100
**9a**	*Cordia myxa* L.	Fruits	1.7	>100
**9b**	*Cordia myxa* L.	Flowering branches	-6.4	>100
**10**	*Fumaria parviflor* *a* Lam.	Leaving branches	6.9	>100
**11**	*Hedera helix *L.	Aerial part	5.4	>100
**12**	*Plantago psyllium* L.	Seeds	12.3	>100
**13**	*Portulaca oleracea* L.	Seeds	-51.1	>100
**14**	*Rosa damascena* Mill.	Flowers	57.7	>100
**15a**	*Viola odorata* L.	Flowers	-110.7	>100
**15b**	*Viola odorata* L.	Whole plant	-81.4	>100
**16**	*Ziziphus jujuba* Mill.	Fruits	-55.3	>100
**17**	5-Fluorouracil	_	_	0.03
**18**	Chloroquine	_	100	>100

NA: not applicable.

## Results and Discussion

The results of the cytotoxicity and the *in-vivo* antiplasmodial of plant extracts were reported in Table 2. Most of them exhibited no significant cytotoxicity. The *Convolvulus scammonia* L. extract was found to be cytotoxic. Twenty extracts were prepared from the selected parts of the sixteen plants species. Eleven extracts (55%) showed *in-vivo *antiplasmodial activity. *Rosa damascena* Mill. showed significant suppression of parasitemia (57.7%). Three plants, *Carthamus tinctorius* L., *Cotoneaster nummularia* Fisch. & Mey. and *Cassia angustifolia* Vahl. showed moderate antiplasmodial activity. *Rosa damascena* Mill. commonly known as rose having several pharmacological properties including anti-HIV, antibacterial, antioxidant, antitussive, hypnotic, anti-diabetic and relaxant effect on tracheal chains have been reported for this plant. Several components were isolated from flowers, petals and hips (seed-pot) of *R. damascena* including terpenes, glycosides, flavonoids, and anthocyanins. This plant contains carboxylic acid, myrcene, vitamin C, kaempferol and quarcetin. Flowers also contain a bitter principle, tanning matter, fatty oil and organic acids. The essential oil of *R.* *damascena*, contains eighteen compounds represented more than 95% of the total oil. The identified compounds were; β-citronellol (14.5-47.5%), nonadecane (10.5-40.5%), geraniol (5.5-18%), andnerol and kaempferol were the major components of the oil. Analyses of rose absolute showed that phenyl ethylalcohol(78.38%), citrenellol (9.91%), nonadecane (4.35%) and geraniol (24-26). In traditional medicine of Iran, *R. damascena *Mill. was used to treat depression, headache, strengthening the heart, skin problems, wounds and quartan fever (16).

The main goal of this work was to investigate the potential antiplasmodial properties of some plants used in traditional medicine of Iran against *nobeh* *fever* and/ or fever. Among sixteen plant species, only three of them (*Althaea officinalis* L.*, Cichorium intybus *L. and *Cordia myxa* L.) have been previously investigated for their antimalarial activity (27-30). So, this is the first report of the antiplasmodial properties of the plants. The next step will be to phytochemical investigation and survey on the mode of action.
